# Evaluation and multi-institutional validation of a novel urine biomarker lncRNA546 to improve the diagnostic specificity of prostate cancer in PSA gray-zone

**DOI:** 10.3389/fonc.2022.946060

**Published:** 2022-08-12

**Authors:** Fei Liu, Xiaolei Shi, Fangming Wang, Sujun Han, Dong Chen, Xu Gao, Linhui Wang, Qiang Wei, Nianzeng Xing, Shancheng Ren

**Affiliations:** ^1^ Department of Urology, National Cancer Center/National Clinical Research Center for Cancer/Cancer Hospital, Chinese Academy of Medical Sciences and Peking Union Medical College, Beijing, China; ^2^ Department of Urology, Shanghai Changhai Hospital, Shanghai, China; ^3^ Department of Urology, West China Hospital, Chengdu, China; ^4^ Department of Urology, Shanghai Changzheng Hospital, Shanghai, China

**Keywords:** prostatic neoplasms, biomarkers, urine, long non-coding RNA, PCA3

## Abstract

**Background and objectives:**

Prostate specific antigen (PSA) is currently the most commonly used biomarker for prostate cancer diagnosis. However, when PSA is in the gray area of 4-10 ng/ml, the diagnostic specificity of prostate cancer is extremely low, leading to overdiagnosis in many clinically false-positive patients. This study was trying to discover and evaluate a novel urine biomarker long non-coding RNA (lncRNA546) to improve the diagnostic accuracy of prostate cancer in PSA gray-zone.

**Methods:**

A cohort study including consecutive 440 participants with suspected prostate cancer was retrospectively conducted in multi-urology centers. LncRNA546 scores were calculated with quantitative real-time polymerase chain reaction. The area under the receiver operating characteristic curve (AUROC), decision curve analysis (DCA) and a biopsy-specific nomogram were utilized to evaluate the potential for clinical application. Logistic regression model was constructed to confirm the predictive power of lncRNA546.

**Results:**

LncRNA546 scores were sufficient to discriminate positive and negative biopsies. ROC analysis showed a higher AUC for lncRNA546 scores than prostate cancer antigen 3 (PCA3) scores (0.78 vs. 0.66, p<0.01) in the overall cohort. More importantly, the AUC of lncRNA546 (0.80) was significantly higher than the AUCs of total PSA (0.57, p=0.02), percentage of free PSA (%fPSA) (0.64, p=0.04) and PCA3 (0.63, p<0.01) in the PSA 4-10 ng/ml cohort. A base model constructed by multiple logistic regression analysis plus lncRNA546 scores improved the predictive accuracy (PA) from 79.8% to 86.3% and improved AUC results from 0.862 to 0.915. DCA showed that the base model plus lncRNA546 displayed greater net benefit at threshold probabilities beyond 15% in the PSA 4-10 ng/ml cohort.

**Conclusion:**

LncRNA546 is a promising novel biomarker for the early detection of prostate cancer, especially in the PSA 4-10 ng/ml cohort.

## Introduction

Prostate cancer (PCa) is the second most frequently diagnosed cancer and the fifth leading cause of cancer death in men worldwide (1). Although its incidence is much lower in China than in Western countries, the PCa incidence is increasing sharply at a rate that ranks it as the malignancy with the fastest increasing incidence (2). The five-year survival rate for localized or regional PCa is approximately 100%, while that of metastatic PCa is only 28% (3). Hence, considerable effort is being made to increase the PCa detection rate at early stages. The serum prostate-specific antigen (PSA) test has contributed to a huge reduction in mortality from prostate cancer (4, 5). Nevertheless, owing to the low specificity of PSA for predicting PCa risk, false-positive PSA test results have led to many unnecessary biopsies and undue socioeconomic burden. Currently, considerable effort is being devoted to exploring more sensitive and specific biomarkers that can assist or replace the PSA test (6–9).

Non-coding RNA longer than 200 nucleotides is termed as long non-coding RNA (lncRNA). Functional characterization and experimental validation have shown that some lncRNA play a major role in disease progression (10). Recent studies suggest lncRNAs play an important role in multiple malignancies including prostate cancer (11). PCa risk and initiation is influenced by lncRNAs, as well as cancer cell proliferation, tumor suppression and treatment resistance (12). Research focusing on lncRNAs, such as prostate cancer antigen 3 (PCA3) (13) and metastasis associated lung adenocarcinoma transcript 1 (MALAT1) (14), may improve current screening techniques to identify patients at risk for PCa.

RNA-seq technology is a far more precise approach for qualitative and quantitative lncRNA discovery and measurement than any other method. Using RNA-seq data from a previous study (15), we analyzed the transcriptomes of 65 pairs of PCa and matched adjacent normal tissue samples. We selected and validated in urine samples and a novel long non-coding RNA (lncRNA546) was found to be promising in predicting the outcome of prostate biopsy.

In this study, we evaluated the diagnostic value of urinary lncRNA546 and compared it with that of PCA3 or PSA levels to predict prostate biopsy outcomes in a multicenter cohort. We also built a predictive model and a lncRNA546-based nomogram to facilitate the diagnosis of PCa in clinical practice.

## Materials and methods

### Patients and study design

A cohort study including consecutive 440 patients was retrospectively conducted in four urologic centers with the approval of research ethics committee (Shanghai Changhai hospital, CHEC-2012-195). Data were retrospectively collected from patients scheduled for prostate biopsy due to elevated serum PSA (≥4 ng/ml) and/or abnormal digital rectum examination (DRE). Patients were excluded from the study if they had a history of PCa, urinary tract infection, other known tumors or were receiving medical therapy that could affect serum PSA levels. Moreover, urine samples were collected from patients with bladder cancer (BCa, 35 cases), renal cancer (RCa, 29 cases), benign prostate hyperplasia (BPH, 36 cases), and prostate cancer (PCa, 29 cases) and from age-matched healthy individuals (20 cases). Informed consent was obtained from all patients.

Study design: In the discovery stage, 248 patients from one institution (Changhai Hospital) were included to assess the lncRNA546 diagnostic ability among the overall cohort and separately in the PSA 4-10 ng/ml cohort. Subsequently, in the validation stage, 192 patients from other three centers were assessed to validate the diagnostic ability of lncRNA546. Finally, all patients were included to constructed logistic regression model and nomogram in order to evaluate the clinical application potential ability.

### LncRNA546 identification and selection

Initially, we identified 480 differentially expressed PCa-associated lncRNA transcripts from RNA-seq data (15) (whole transcriptome sequencing data has been deposited in The European Genome-phenome Archive (EGAS00001000888)). Through screening by literature review, four lncRNAs were selected and validated in urine samples and a novel long non-coding RNA (lncRNA546) was found to be promising in predicting the outcome of prostate biopsy.

### Specimen collection and processing

First-catch post-massage (PM) urine specimens from patients suspected of having prostate cancer were obtained before prostate biopsy following an attentive DRE (3 strokes per lobe) using a validated standard operating procedure (9), and urine samples from patients with BCa and RCa, as well as age-matched healthy individuals, were collected after the DRE procedure. The samples were immediately cooled on ice and processed within 2 hours. To separate sediment from supernatant, the urine samples were centrifuged at 4000 rpm for 15 minutes at 4°C, and then the pellets were washed twice with ice-cold PBS. The urine sediment was dissolved in TRIzol reagent (Invitrogen) and stored at -80°C before further use. All urine sediments samples were processed according to standard operating procedure (SOP) of Chinese Prostate Cancer Consortium (CPCC), and then delivered to Changhai Hospital Urology Laboratory by the same set of cold-chain transportation for further processing (14).

### WTA and quantitative qRT-PCR analysis

The total RNA of the samples was extracted using TRIzol reagent. Sense-strand cDNA was synthesized and amplified using the TransPlex Complete Whole Transcriptome Amplification Kit (WTA2, Sigma–Aldrich, St. Louis, MO) according to the manufacturer’s instructions. Quantitative reverse transcription polymerase chain (qRT-PCR) was performed using THUNDERBIRD™ SYBR^®^ qPCR Mix (TOYOBO: QPS201) and a Step One Plus system (Applied Biosystems, USA). The amplification conditions were as follows: 95°C for 10 min, followed by 40 cycles of 95°C for 15s and 60°C for 60s. The primer sequences for the qRT-PCR were as follows: PSA-forward GTCTGCGGCGGTGTTCTG, PSA-reverse TGCCGACCCAGCAAGATC; PCA3-forward GAGAACAGGGGAGGGAGAG, PCA3-reverse CATGTCGCTGGCCTCTCAA; lncRNA546-forward TCCTCCTAAGCCGTATCCCATCTG, lncRNA546-reverse CCAGGTGAGTTGAACAGTCCGATT. The data were analyzed using StepOne Software v2.1 (Applied Biosystems, USA). To eliminate samples with insufficient prostate cell collection, samples with PSA cycle threshold (Ct) values over 28 were excluded. The lncRNA546 score was calculated based on the formula lncRNA546 mRNA/PSA mRNA × 100 = 2^Ct(PSA)-Ct (lncRNA546)^ × 100, and the PCA3 score was calculated as previously described (16). All experiments were performed in triplicate.

### Statistical analysis

Continuous variables were compared by Student’s t-test for variables with normal distribution, or the Mann–Whitney U test for variables without normal distribution; categorical variables were compared by Pearson’s Chi-square test or Fisher’s exact test. The area under the curve (AUC) of receiver operator characteristic (ROC) analysis was used to assess diagnostic ability. Univariate and multivariate logistic regression analyses were used to determine the presence of PCa from the biopsy data. Decision curve analysis (DCA) was applied to assess clinical performance and to compare prediction models. Bootstrap resampling was used for internal validation by a biopsy-specific nomogram. Two-sided p-values <0.05 were considered statistically significant. Analyses were performed using the software packages SPSS v.19.0 (SPSS, IL USA), MedCalc v.11.4 (MedCalc Software bvba, Mariakerke, Belgium), Stata v.12.0 (StataCorp, Texas, USA) and R v.3.2.2 (R Foundation for Statistical Computing, Vienna, Austria).

## Results

### Discovery of the diagnostic value for urinary lncRNA546 score

The urinary abundance of lncRNA546 was analyzed in patients with PCa, BCa, RCa or BPH, as well as in age-matched healthy individuals (**Figure S1**). LncRNA546 could be detected in all five groups, and patients with PCa showed higher expression of lncRNA546 than all of the other participants, suggesting that lncRNA546 is correlated with prostate cancer.

**Figure 1:**
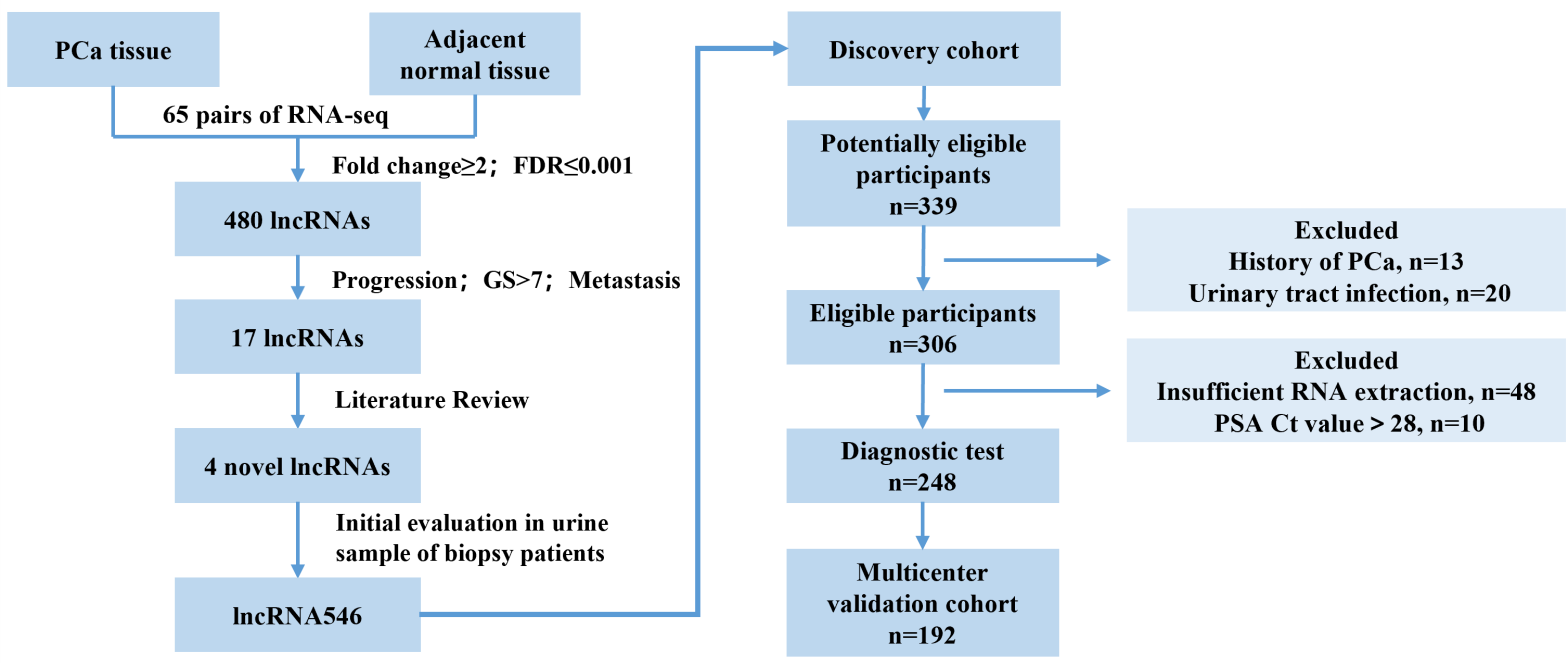
Flow diagram of evaluation and multi-institutional validation for the diagnostic value of urinary lncRNA546 in prostate cancer. PCa, prostate cancer. FDR, false discovery rate. GS, Gleason score.

This study flow diagram was as shown in **Figure 1**. After initial evaluation in urine samples, the diagnostic value of lncRNA546 was explored among patients referred for a prostate biopsy in Changhai Hospital. Of 306 cases subjected to analysis, 48 cases were excluded due to insufficient RNA extraction, and 10 cases were excluded based on PSA Ct values that exceeded 28. As shown in the baseline patient characteristics (**Table 1**), PCa-associated risk factors, including age, PSA, prostate volume, %fPSA, DRE, PSAD, PCA3 score and lncRNA546 score, were all statistically significant in their ability to differentiate prostate cancer from negative biopsies in the overall cohort.

**Table 1:** Prostate cancer-related risk factors in the discovery cohort.

Parameter	Overall cohort				PSA 4-10 ng/ml cohort			
	Entire	Negative	Positive	p-value	Entire	Negative	Positive	p-value
Age, yr				<0.001^*^				0.029^*^
No. pts (%)	248 (100.0)	149 (60.1)	99 (39.9)		93 (100.0)	73 (78.5)	20 (21.5)	
Mean	66.7	65.3	68.8		65.7	64.8	68.9	
SD	7.0	7.0	6.3		7.3	7.2	7.0	
tPSA, ng/ml				<0.001^#^				0.340^#^
No. pts (%)	248 (100.0)	149 (60.1)	99 (39.9)		93 (100.0)	73 (78.5)	20 (21.5)	
Median	11.4	9.5	23.1		7.3	7.2	7.6	
IQR	7.6-23.1	7.1-14.7	10.6-63.8		6.5-8.4	6.5-8.3	6.2-8.9	
Volume, cm^3^				<0.001^#^				0.001^#^
No. pts (%)	248 (100.0)	149 (60.1)	99 (39.9)		93 (100.0)	73 (78.5)	20 (21.5)	
Median	46.0	52.2	39.9		50.8	53.6	39.9	
IQR	35.0-61.2	39.5-67.0	31.5-50.9		38.2-63.1	41.3-69.8	32.2-49.2	
%fPSA				0.001^#^				0.030^#^
No. pts (%)	213 (100.0)	133 (62.4)	80 (37.6)		85 (100.0)	68 (80.0)	17 (20.0)	
Median	0.15	0.16	0.12		0.17	0.17	0.12	
IQR	0.09-0.20	0.11-0.21	0.07-0.18		0.12-0.21	0.13-0.23	0.10-0.18	
Suspicious DRE				<0.001^§^				0.007^&^
No. pts	248	149	99		93	73	20	
No.%	61 (24.6)	19 (12.8)	42 (42.4)		16 (17.2)	8 (11.0)	8 (40.0)	
PSAD				<0.001^#^				<0.001^#^
No. pts	248 (100.0)	149 (60.1)	99 (39.9)		93 (100.0)	73 (78.5)	20 (21.5)	
Median	0.26	0.18	0.60		0.15	0.13	0.19	
IQR	0.15-0.60	0.13-0.31	0.31-1.77		0.11-0.20	0.10-0.18	0.17-0.23	
PCA3 Score				<0.001^#^				0.077^#^
No. pts (%)	248 (100.0)	149 (60.1)	99 (39.9)		93 (100.0)	73 (78.5)	20 (21.5)	
Median	74.3	57.8	97.2		61.0	57.8	76.0	
IQR	25.7-140.4	19.0-112.0	49.9-198.7		18.2-99.7	12.8-97.4	51.9-118.7	
lncRNA 546				<0.001^#^				<0.001^#^
No. pts (%)	248 (100.0)	149 (60.1)	99 (39.9)		93 (100.0)	73 (78.5)	20 (21.5)	
Median	59.0	42.9	105.0		51.0	42.9	95.8	
IQR	24.1-105.4	17.4-69.0	54.7-201.9		21.7-95.4	16.1-79.9	67.2-176.8	
Gleason sum, No. (%)								
≤6			22 (22.2)				6 (30.0)	
7			32 (32.3)				9 (45.0)	
≥8			45 (45.5)				5 (25.0)	

Yr, year; pts, percentages; SD, standard deviation; IQR, interquartile range; PSA, prostate-specific antigen; tPSA, total PSA; %fPSA, percent free PSA; PSAD, PSA density; DRE, digital rectal examination.

*Student’s t test.

^#^Mann-Whitney U test.

^§^Pearson Chi-square test.

^ð^Continuity-adjusted chi-square test.

**Figure 2:**
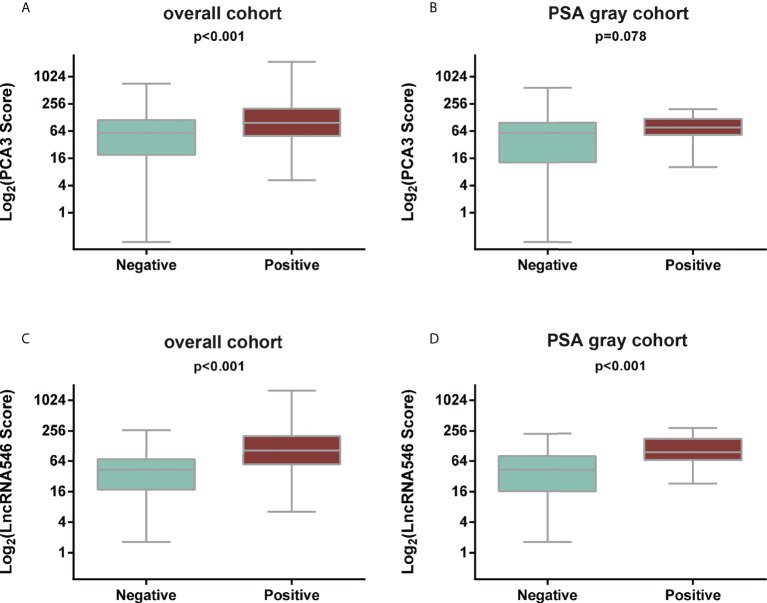
Comparison of PCA3 and LncRNA546 scores between positive and negative biopsies in two cohorts. **(A)** PCA3 score in the overall cohort. **(B)** PCA3 score in the PSA gray area cohort. **(C)** LncRNA546 score in the overall cohort. **(D)** LncRNA546 score in the PSA gray area cohort.

Notably, lncRNA546 showed a higher score in the positive biopsy outcome group than in the negative group in the overall cohort. Likewise, the lncRNA546 score could be used to discriminate positive from negative biopsies in the PSA gray area (**Figure 2**).

**Figure 3:**
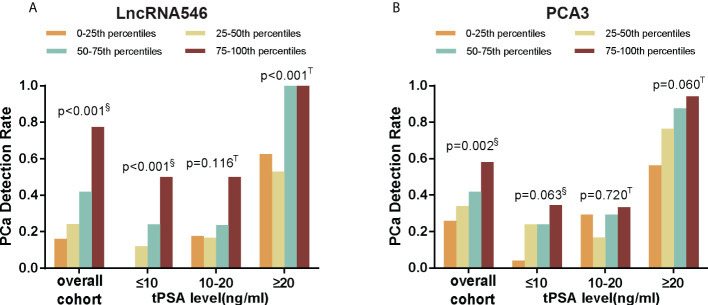
PCa detection rate of different score percentiles in overall, tPSA ≤10, 10-20, and ≥20 ng/ml cohorts. **(A)** LncRNA546 score. **(B)** PCA3 score. ^§^Pearson Chi-square test; ^T^ Fisher’s exact test.

The results above indicate that lncRNA546 has potential as a useful urine biomarker for predicting the diagnostic outcome of prostate biopsies. Therefore, we continued to investigate the association between the lncRNA546 score and the detection rate of PCa. As shown in **Figure 3**, as the lncRNA546 score increased, the detection rate of PCa increased sharply in each of the cohorts (the overall cohort and the PSA 4-10 ng/ml and PSA >20 ng/ml cohorts). By contrast, PCA3 showed a significantly higher detection rate only in the overall cohort.

**Figure 4:**
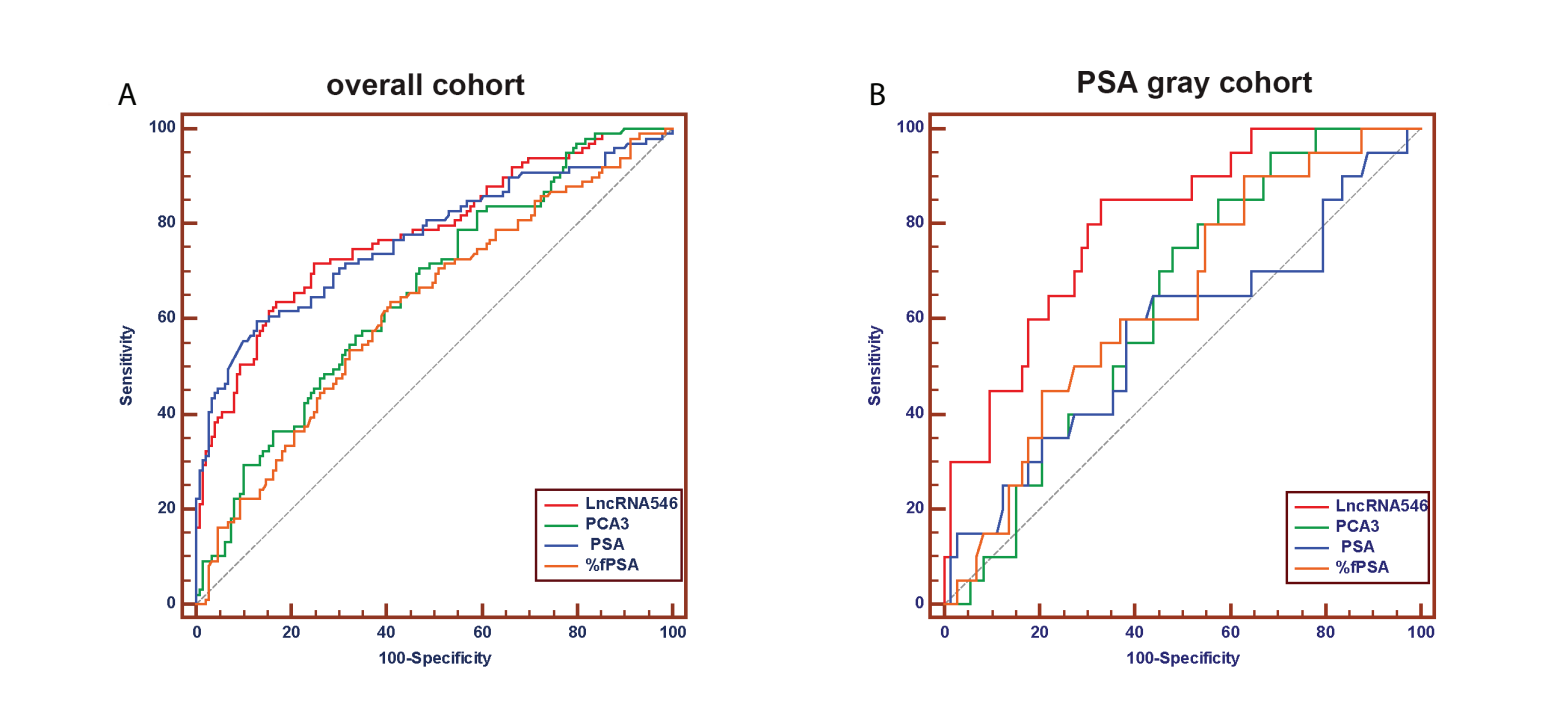
Receiver operating characteristic (ROC) curve analysis for evaluating the diagnostic performance of LncRNA546 score compared with tPSA, %fPSA, PCA3. **(A)** in the overall cohort. **(B)** in the PSA gray area cohort.

To evaluate the predictive power of lncRNA546, the AUC of the ROC was calculated for the biomarkers under investigation (**Figure 4**). The AUC of lncRNA546 indicated that use of this biomarker was more effective than the PSA test (0.80 vs. 0.57, p=0.02), %fPSA (0.80 vs. 0.64, p=0.04) and PCA3 (0.80 vs. 0.63, p<0.01) in the PSA 4-10 ng/ml cohort. Interestingly, lncRNA546 score was found to be much higher in the high-grade prostate cancer (HGPCa, Gleason score≥7) group than the low-grade prostate cancer (LGPCa, Gleason score ≤ 6) group (108.8 vs 65.7, p=0.027) (**Figure S2**). These data suggested that lncRNA546 might be correlated with Gleason score and probably the prognosis of PCa as well. More researches are guaranteed to investigate the prognostic value of lncRNA546 in the next few years.

As shown in **Table 2**, our data indicated that the diagnostic accuracy of lncRNA546 exceeded that of traditional biomarkers, such as %fPSA and PCA3, in the PSA gray area. When we apply a lncRNA546 cutoff value of 58, the sensitivity and specificity of lncRNA546 are superior to those of %fPSA and PCA3. These results suggest that prostate cancer can be more accurately detected when using a lncRNA546 score cutoff of 58 than when using a %fPSA cutoff value of 0.16 or a PCA3 cutoff value of 41 in the PSA gray area cohort and that unnecessary biopsies could thus be avoided. These data suggest that lncRNA546 could be a promising biomarker for PSA test results between 4 and 10 ng/ml.

**Table 2:** Comparison of %fPSA, PCA3 and lncRNA546 with respect to specific diagnostic performance at the recommended cutoff values in the PSA gray area cohort.

Biomarker	Cutoff	Sensitivity, % (95% CI)	Specificity, % (95% CI)	PCa detected, %	Unnecessary biopsies avoided, %
%fPSA	0.16	60.0 (36.1-80.9)	60.3 (48.7-69.3)	12/20 (60.0)	44/73 (60.3)
PCA3	41	85.0 (62.1-96.8)	42.5 (31.0-54.6)	17/20 (85.0)	31/73 (42.5)
lncRNA546	58	85.0 (62.1-96.8)	67.1 (55.1-77.7)	17/20 (85.0)	49/73 (67.1)

PCa, prostate cancer; %fPSA, percent free PSA.

### Multicenter validation of the diagnostic value of urinary lncRNA546 score

**Figure 5:**
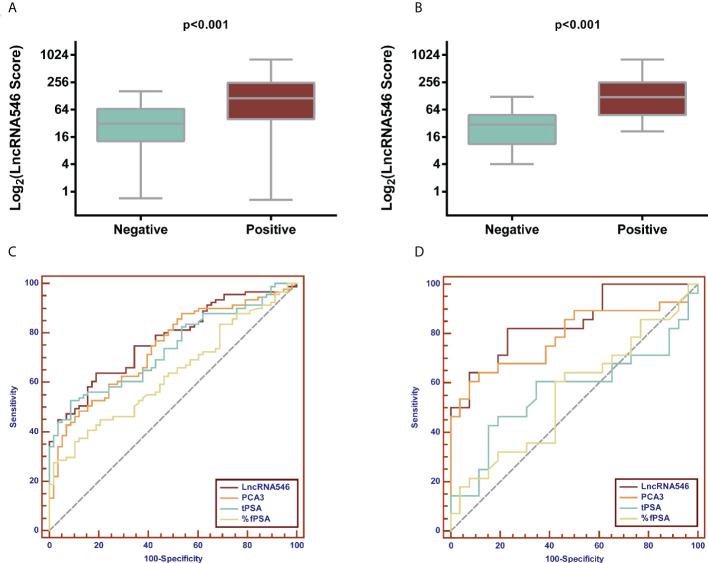
Validation of diagnostic performance of LncRNA546 from multicenter data. LncRNA546 score between positive and negative biopsy groups in the overall cohort **(A)** and in the PSA gray area cohort **(B)**. ROC curves for LncRNA546 compared with PCA3, tPSA and %fPSA in the overall cohort **(C)** and PSA gray area cohort **(D)**.

Based on our results suggesting that lncRNA546 could be a novel promising biomarker for PCa diagnosis, we further validated these results with a discovery cohort of 192 patients from three other urological centers. These patients were analyzed to evaluate the ROC and the levels of differential expression between PCa and BPH patients (**Figures 5**, **S3**). Urine lncRNA546 measurements were significantly higher in patients with PCa relative to those with BPH. Moreover, lncRNA546 showed reliable results at predicting prostate biopsy outcomes in the overall cohort with AUC=0.77. Additionally, the AUC values observed in the PSA 4-10 ng/ml cohort for lncRNA546 were consistently higher than those observed for %fPSA (0.80 vs. 0.58, p=0.02) or PSA (0.80 vs. 0.60, p=0.03).

### Logistic regression model (LRM) and decision curve analysis (DCA) based on lncRNA546 score

After confirming the predictive power of lncRNA546, the lncRNA546 score was utilized to construct a logistic regression model with other risk factors. First, a univariate logistic regression model was applied to measure the odds ratio (OR), predictive accuracy (PA) and AUC of each individual risk factor (**Table S1**). LncRNA546 displayed higher PA (73.8%, 82.8%) and AUC (0.780, 0.798) values than most other variables in the overall cohort, as well as in the PSA 4-10 ng/ml cohort. Next, a multivariate logistic regression model was constructed according to the p-value with a base model that included age, volume, %fPSA, and DRE with PSA in the overall cohort and omitted tPSA in the PSA 4-10 ng/ml cohort (**Table 3**). The results of this analysis revealed that the base model plus lncRNA546 score improved the PA from 79.8% to 86.3% and improved the AUC from 0.862 to 0.915 in the overall cohort, which were markedly higher than the PA and AUC of PCA3. In the PSA gray zone cohort, lncRNA546 enhanced the PA by 2.1% and increased the AUC by 0.06, although this difference did not reach statistical significance. PCA3 contributed almost no benefit to the model. The ROC curve of each model is presented to illustrate the benefit of including lncRNA546 (**Figure S4**).

**Table 3:** Multivariable logistic regression models to predict prostate cancer.

Variables	Overall cohort			PSA 4-10 ng/ml cohort		
	Base Model^α^	Base Model Plus PCA3 Score	Base Model Plus lncRNA546 Score	Base Model^β^	Base Model Plus PCA3 Score	Base Model Plus lncRNA546 Score
	OR (95% CI);p	OR (95% CI);p	OR (95% CI);p	OR (95% CI);p	OR (95% CI);p	OR (95% CI);p
Age	1.1041 (1.0469-1.1643);<0.001	1.1031 (1.0449-1.1646);<0.001	1.0960 (1.0305-1.1656);0.004	1.2059 (1.0733-1.3548);0.002	1.2036 (1.0698-1.3541);0.002	1.2042 (1.0484-1.3831);0.009
Volume	0.9664 (0.9469-0.9863);0.001	0.9648 (0.9450-0.9849);0.001	0.9589 (0.9355-0.9830);0.001	0.9271 (0.8779-0.9792);0.007	0.9250 (0.8752-0.9777);0.006	0.9250 (0.8655-0.9887);0.022
%fPSA	0.0054 (0.0001-0.4054);0.018	0.0124 (0.0002-0.8877);0.044	0.0225 (0.0002-2.7983);0.123	0.0000 (0.0000-7.4791);0.103	0.0001 (0.0000-16.3537);0.132	0.0000 (0.0000-8.3132);0.090
DRE	3.0554 (1.3784-6.7728);0.006	3.1385 (1.4050-7.0108);0.005	4.3080 (1.7659-10.5096);0.001	5.6798 (1.1984-26.9188);0.029	6.0464 (1.2251-29.8405);0.027	17.2594 (1.9714-151.1032);0.010
tPSA	1.0531 (1.0283-1.0784);<0.001	1.0508 (1.0259-1.0763);<0.001	1.0543 (1.0272-1.0822);<0.001	–	–	–
PCA3 score	–	1.0030 (1.0000-1.0061);0.049	–	–	1.0038 (0.9948-1.0128);0.413	–
lncRNA 546 score	–	–	1.0182 (1.0110-1.0253);<0.001	–	–	1.0246 (1.0094-1.0401);0.001
PA (%)	79.8	79.4	86.3	87.1	87.1	89.2
Increment PA (%)	–	-0.4	6.5	–	0.0	2.1
AUC (95%CI)	0.862 (0.817-0.908)	0.867 (0.822-0.912)	0.915 (0.879-0.951)	0.864 (0.780-0.949)	0.865 (0.779-0.951)	0.927 (0.862-0.992)
Increment AUC; p	–	0.005;0.496	0.048;0.004	–	0.001;0.934	0.062;0.054

CI, confidence interval; AUC, area under receiver operating characteristic curve; PA, predictive accuracy; %fPSA, percent free PSA; DRE, digital rectal examination.

^α^The base model consists of age, volume, %fPSA, DRE, tPSA.

^β^The base model consists of age, volume, %fPSA, DRE.

**Figure 6:**
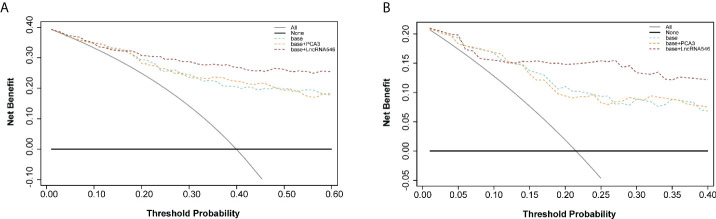
Decision curve analysis of predicting PCa with base, base+PCA3 score, base+LncRNA546 score model. **(A)** in the overall cohort. **(B)** in the PSA gray area cohort.

To further substantiate our findings, a decision curve analysis was applied to evaluate the clinical value of lncRNA546. LncRNA546 performance demonstrated a high net benefit, as its use led to a net reduction in avoidable biopsies relative to commonly acknowledged biomarkers, especially in the PSA 4-10 ng/ml cohort (**Figure 6**). Furthermore, the base model plus lncRNA546 displayed greater net benefit and more net reduction in avoidable biopsies than those found for the other two models at threshold probabilities beyond 15% in the PSA 4-10 ng/ml cohort (**Figure 6**, **Table S2**). **Table S3** reveals that the base model plus lncRNA546 could be used to prevent more unnecessary biopsies, as well as a decrease in missed PCa cases, without omitting any high-grade prostate cancer (HGPCa) above 20% threshold probability in the PSA 4-10 ng/ml cohort. These findings demonstrate that lncRNA546 is highly efficient either alone or in conjunction with established biomarkers, especially in the PSA gray area cohort.

### Development and internal validation of a nomogram including lncRNA546 to predict prostate cancer biopsy outcome

**Figure 7:**
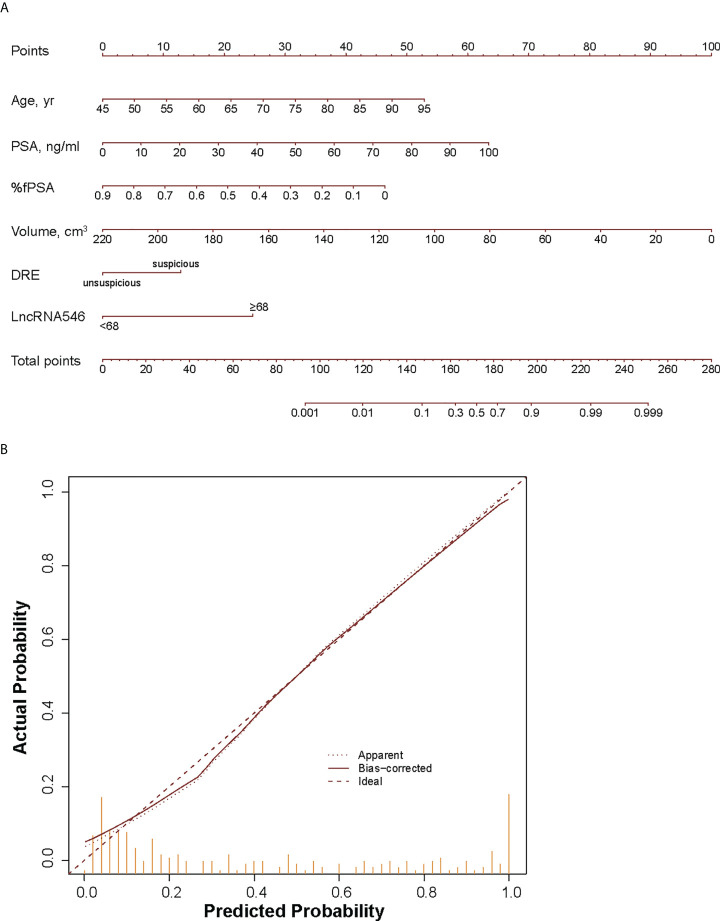
Development **(A)** and calibration curve **(B)** of LncRNA546 nomogram for early diagnosis of PCa. yr, year; %fPSA, percent free PSA; DRE, digital rectal examination.

Because the logistic regression model plus lncRNA546 performed well at predicting the biopsy outcome, we built a nomogram with an associated calibration curve based on our model (**Figure 7**). The calibration curve figure suggests that the bias-corrected model is in close agreement with the 45° line, which indicates near-perfect prediction. Accordingly, this nomogram could be used to help clinical urologists conveniently estimate the probability that a patient has prostate cancer based on risk factors.

## Discussion

Serum PSA is a good indicator to guide prostate biopsy when values are higher than 10 ng/ml. Although the introduction of serum PSA testing has greatly improved early detection rates and prediction of disease advancement, PSA between 4 and 10 ng/ml is still generally regarded as a “gray zone” because 60%-75% of men with PSA values in this range do not have PCa, which could cause this cohort to undergo unnecessary biopsies (17). Our study found a significant AUC decrease in the predictive power of PSA from 0.770 (0.706-0.833) in the overall cohort to 0.570 (0.417-0.723) in the PSA gray zone cohort. Notably, only 21.5% of men with PSA levels in this range displayed a positive biopsy outcome, which suggests that 78.5% of these men received unnecessary invasive biopsies and possibly suffered consequent complications. Measurement of the free-to-total PSA (f/tPSA) ratio is considered the most widely used reflex test and performs better than PSA levels alone. Moreover, this ratio can distinguish between benign prostate disease and malignancy in men with PSA levels from 4 to 10 ng/ml (18–21). We observed consistent results in which f/tPSA outperformed PSA (AUC 0.638 vs. 0.570, p=0.47) in the PSA gray zone cohort, although this difference did not reach statistical significance. More importantly, the novel urine biomarker we propose, lncRNA546, proved to be a more efficient predictive tool than %fPSA (AUC 0.798 vs. 0.638, p=0.08) in men in the PSA 4-10 ng/ml cohort, although the p-value was not significant, which could be due to the small number of patients analyzed. Further large-scale studies are warranted to verify comparisons among different tests for patients in the PSA gray zone.

To overcome the limitations of PSA levels and to improve PCa diagnostic accuracy, multiple biomarkers have been proposed for men undergoing prostate biopsy. The most promising biomarker, PCA3, has attracted considerable attention and appears to offer advantages over PSA levels (13, 22). Several recent studies have demonstrated the outstanding predictive power of PCA3. In a large multi-institutional data set of 809 men at risk of PCa, Chun et al. reported an AUC of 0.68 for PCA3 for mixed biopsies (23). Similarly, Deras et al. reported an area under the ROC curve of 0.69 for PCA3 in mixed biopsies (24). Moreover, for men undergoing repeat biopsies, Marks et al. and Haese et al. reported AUC values for PCA3 of 0.68 and 0.66, respectively (25, 26). Wang et al. first reported the diagnostic value of PCA3 in a Chinese population (16); in their report, PCA3 scores discriminated positive from negative prostate biopsy results but did not correlate with the aggressiveness of PCa. Before our study, most studies focused primarily on repeat or mixed biopsy settings, and convincing evidence for the predictive power of urine PCA3 in Chinese men is scarce. In the present multicenter study, a comparable AUC value for PCA3 was observed to predict initial biopsy outcome with a value of 0.659, which suggests the efficacy of urine PCA3 analyses in a Chinese cohort. We used qRT-PCR to quantify PCA3 in urine sediment (26, 27). The results achieved using this approach were consistent with a previous report of PCA3 diagnostic values (16) and help to broaden the clinical applicability of PCA3 screening. In addition, we demonstrated for the first time that lncRNA546 has more reliable predictive value than PCA3 (AUC 0.780 vs. 0.659, p<0.01). Applying a lncRNA546 score cutoff of 68, the sensitivity and specificity were 71.7% and 75.2%, respectively. Furthermore, adding lncRNA546 to the base model in our logistic regression analysis increased its PA by 6.5%, while including PCA3 resulted in a 0.4% decrease in the PA of the model.

While a number of studies have demonstrated the clinical application of lncRNAs in prostate cancer and in other cancer types, the specific biological roles of lncRNAs remain to be elucidated. The lncRNA PCAT-14 expression was associated with metastatic progression, overall survival and disease-specific mortality in prostate cancer (28). MALAT-1 was reported to maintain prostate tumorigenicity and involved in prostate cancer progression (29). Similarly, the lncRNA546 has been reported to enhance cell proliferation and promote migration and invasion in prostate cancer (30).

It is generally accepted that there is an unmet need to improve information obtained from PSA testing for PCa screening and early detection due to its lack of specificity. The lack of specificity of PSA screening results in numerous unnecessary biopsies and overtreatment, as well as substantial corresponding costs and psychological stress (31, 32). However, efforts to augment the specificity of the current detection strategy have been offset by a simultaneous reduction in sensitivity, and the use of PSA as a single biomarker thus carries major limitations. Therefore, how to balance the trade-off between avoiding unnecessary prostate biopsies and missing aggressive PCa is a major challenge faced by urologists and patients. The decision curve analysis in the current study confirmed that both the lncRNA546 score alone and a lncRNA546-based model can help to avoid unnecessary biopsies without significantly increasing the false-negative rate for PCa and HGPCa. Collectively, our results suggest that the lncRNA546 score may be more accurate than f/tPSA ratio, which is the most widely used parameter for PSA gray zone patients.

Prostate volume (PV) data were used to construct a nomogram, but due to inherent differences in clinical data collection at multiple centers, the nomogram only included data from three centers. After bias correction, the accuracy of the nomogram including the novel biomarker lncRNA546 was 86.3% for the prediction of biopsy outcomes, which is remarkably high. Inclusion of lncRNA546 led to a distinct reduction in the number of unnecessary biopsies relative to the base model. Although external validations are needed to confirm our results, the calibration plot appears reliable, and an internal validation of 1000 bootstrap resamples indicates stability of our results.

Despite the encouraging results, this study has several limitations. First, our research relied on a cohort of a relatively small number of patients. Second, prostate cancer was detected in 40.0% of patients in our study, which is much higher than the proportion reported in screening trials (24.5%) (33). Thus, the performance of lncRNA546 reported in our study applies only to an early detection patient cohort instead of a general population of men who are undergoing screening for PCa. Finally, prostate biopsy outcome variability may have been influenced by the lack of a central pathology review. Nevertheless, strict protocols were followed at the four institutions included in our study, and experienced uropathologists confirmed the results.

## Conclusions

Collectively, the lncRNA546 score in post-PM urine proved to be an effective biomarker for predicting the outcome of prostate biopsies in men with elevated PSA and/or abnormal DRE results. In the PSA gray zone in particular, lncRNA546 showed better predictive value for the early diagnosis of prostate cancer than current biomarkers.

## Data availability statement

The original contributions presented in the study are included in the article/**Supplementary Material**. Further inquiries can be directed to the corresponding authors.

## Ethics statement

Study received Shanghai Changhai Hospital institutional review board approval (CHEC-2012-195). The patients/participants provided their written informed consent to participate in this study.

## Author contributions

FL and XS carried out the experiments, participated in data analysis, performed statistical analysis and drafted the manuscript. F-MW, SH, and DC helped performed experiments and collected clinical data, XG, QW, LW, NX, and SR provided clinical samples and clinical data; FL and SR conceived of the study and participated in its design and coordination and helped to draft the manuscript. All authors read and approved the final version of the manuscript.

## Funding

Supported by the National Natural Science Foundation of China (No. 81972400 and No. 32100631), the Young Elite Scientists Sponsorship Program by China Association for Science and Technology (YESS20210056), the Beijing Excellent Talents Program-Youth Backbone Project (No. 2018000032600G393) and the Beijing Hope Run Special Fund of Cancer Foundation of China (No. LC2019B02).

## Conflict of interest

The authors declare that the research was conducted in the absence of any commercial or financial relationships that could be construed as a potential conflict of interest.

## Publisher’s note

All claims expressed in this article are solely those of the authors and do not necessarily represent those of their affiliated organizations, or those of the publisher, the editors and the reviewers. Any product that may be evaluated in this article, or claim that may be made by its manufacturer, is not guaranteed or endorsed by the publisher.
