# Supplementary material for: Evaluation and multi-institutional validation of a novel urine biomarker lncRNA546 to improve the diagnostic specificity of prostate cancer in PSA gray-zone
Source: Front Oncol. 2022 Aug 12;12:946060. doi: 10.3389/fonc.2022.946060 (PMC9411806; doi:10.3389/fonc.2022.946060)
Supplement: Supplementary file 1 [file DataSheet_1.docx]

**Supplementary Materials**

Figure S1. Comparison of lncRNA546 abundance in urine sediments of prostate cancer (PCa), bladder cancer (BCa), renal cancer (RCa), benign prostate hyperplasia (BPH) and healthy patients. ***: P<0.001.


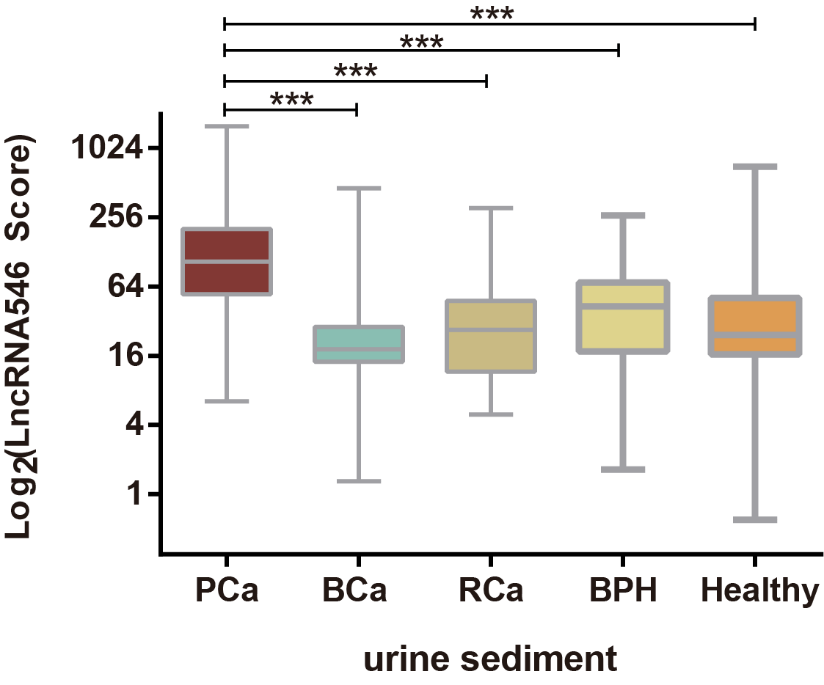


Figure S2. Comparison of lncRNA546 score in healthy, low grade and high grade prostate cancer patients. *: P<0.05; ***: P<0.001


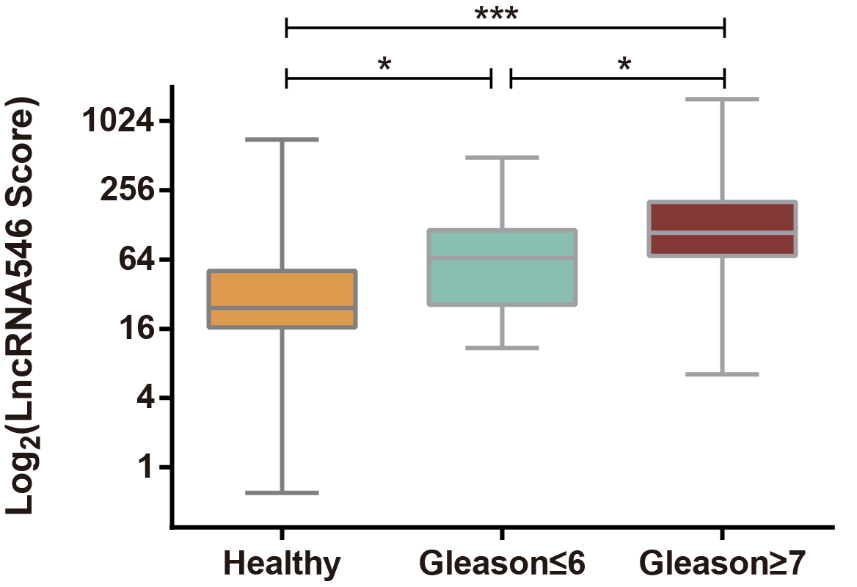


Figure S3. The diagnostic performance of PCA3 in multiple centers. (A) Results for the overall cohort. (B) Results for the PSA gray area cohort.


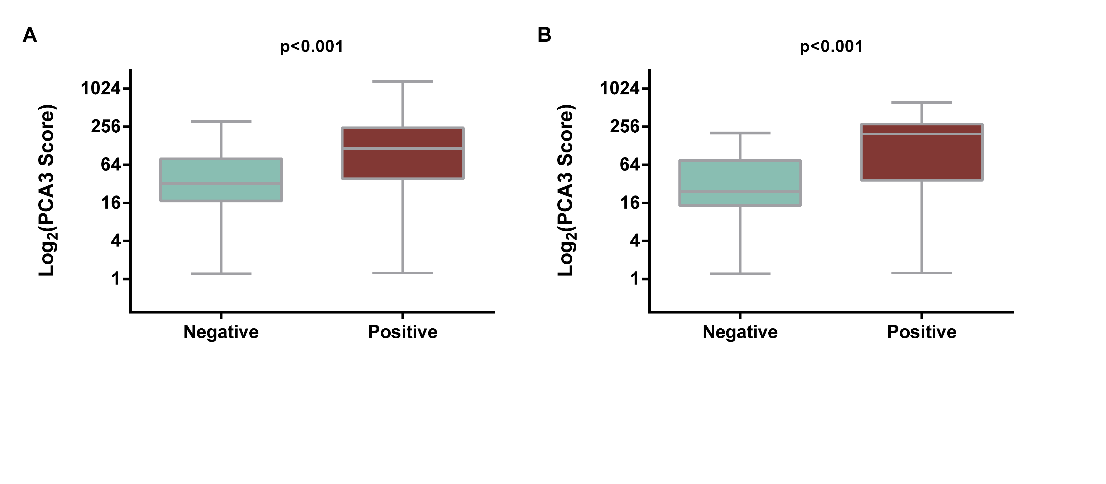


Figure S4. ROC curve analysis for the evaluation and comparison of logistic regression models (base, base+PCA3 and base+lncRNA546). (A) Results for the overall cohort. (B) Results for the PSA gray area cohort.


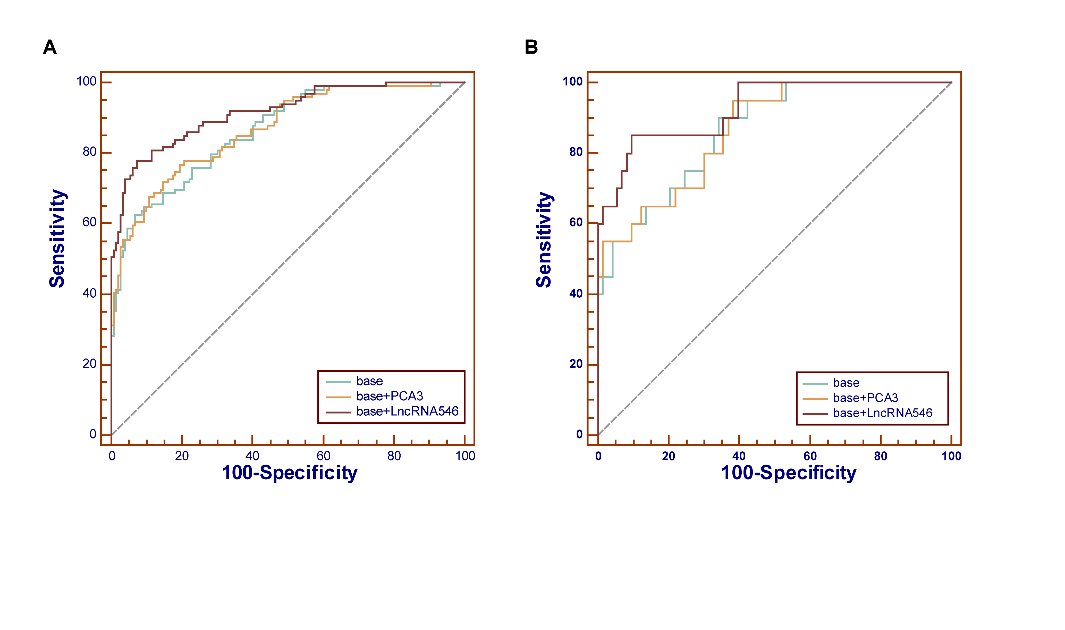


Table S1. Univariate logistic regression models to predict prostate cancer

| Variables | Overall cohort | | | PSA 4-10 ng/ml cohort | | |
| --- | --- | --- | --- | --- | --- | --- |
|  | OR(95% CI);p | PA（%） | AUC (95% CI) | OR (95% CI);p | PA（%） | AUC (95% CI) |
| lncRNA546 score | 1.0173(1.0115-1.0230);<0.001 | 73.8 | 0.780(0.720-0.840) | 1.0202(1.0093-1.0312);0.001 | 82.8 | 0.798(0.696-0.900) |
| PCA3 score | 1.0038(1.0014-1.0061);0.002 | 63.7 | 0.659(0.591-0.727) | 1.0012(0.9957-1.0068);0.671 | 78.5 | 0.629(0.509-0.750) |
| Age | 1.0811(1.0383-1.1257);<0.001 | 62.5 | 0.647(0.578-0.716) | 1.0829(1.0066-1.1785);0.034 | 78.5 | 0.663(0.527-0.799) |
| tPSA | 1.0657(1.0414-1.0906);<0.001 | 74.6 | 0.770(0.706-0.833) | 1.1677(0.8135-1.6760);0.400 | 78.5 | 0.570(0.417-0.723) |
| Volume | 0.9627(0.9474-0.9783);<0.001 | 62.9 | 0.696(0.631-0.762) | 0.9491(0.9165-0.9829);0.003 | 78.5 | 0.739(0.633-0.846) |
| %fPSA | 0.0096(0.0004-0.2446);0.005 | 62.1 | 0.624(0.553-0.695) | 0.0008(0.0000-1.5384);0.065 | 78.5 | 0.638(0.509-0.768) |
| DRE | 5.0416(2.6986-9.4186);<0.001 | 69.4 | 0.648(0.576-0.721) | 5.4167(1.7025-17.2340);0.004 | 78.5 | 0.645(0.496-0.794) |

OR=odds ratio; CI=confidence interval; AUC=area under receiver operating characteristic curve; PA=predictive accuracy; DRE=digital rectal examination; %fPSA=percent free PSA.

Table S2. Comparison of net benefit and net reduction in avoidable biopsies for the three combined models and the treat all strategy in the threshold probability range of 10-40% for the overall cohort and the PSA gray area cohort.

| Threshold probability (%) | | | 10 | 15 | 20 | 25 | 30 | 35 | 40 |
| --- | --- | --- | --- | --- | --- | --- | --- | --- | --- |
| Overall cohort | Net benefit (%) | base model^α^ | 34.8 | 33.1 | 29.3 | 25.5 | 24.5 | 22.4 | 20.6 |
|  |  | base model +PCA3 score | 34.9 | 32.6 | 28.1 | 26.5 | 23.6 | 23.7 | 22.3 |
|  |  | base model+lncRNA546 score | 34.6 | 32.4 | 30.7 | 29.4 | 28.8 | 27.9 | 26.9 |
|  |  | Treat all | 33.2 | 29.3 | 24.9 | 19.9 | 14.2 | 7.6 | -0.1 |
|  | Net reduction in avoidable biopsies (%) | base model^α^ | 14.1 | 21.2 | 17.7 | 16.9 | 24.1 | 27.6 | 31.0 |
|  |  | base model +PCA3 score | 15.3 | 18.5 | 12.9 | 19.8 | 22.0 | 29.9 | 33.7 |
|  |  | base model+lncRNA546 score | 12.5 | 17.2 | 23.4 | 28.6 | 34.1 | 37.7 | 40.5 |
| PSA gray area  cohort | Net benefit (%) | base model^β^ | 16.7 | 13.7 | 11.0 | 9.3 | 8.9 | 8.9 | 6.8 |
|  |  | base model +PCA3 score | 16.9 | 13.2 | 9.4 | 8.2 | 8.9 | 8.9 | 7.5 |
|  |  | base model+lncRNA546 score | 15.5 | 15.2 | 14.8 | 15.4 | 13.4 | 12.2 | 12.2 |
|  |  | Treat all | 12.8 | 7.7 | 1.9 | -4.7 | -12.1 | -20.8 | -30.8 |
|  | Net reduction in avoidable biopsies (%) | base model^β^ | 35.5 | 34.4 | 36.6 | 41.9 | 49.1 | 55.0 | 56.5 |
|  |  | base model +PCA3 score | 36.6 | 31.2 | 30.1 | 38.7 | 49.1 | 55.0 | 57.5 |
|  |  | base model+lncRNA546 score | 24.7 | 43.0 | 51.6 | 60.2 | 59.5 | 61.1 | 64.5 |

^α^The base model consists of age, volume, %fPSA, DRE and tPSA.

^β^The base model consists of age, volume, %fPSA and DRE.

**Table S3. Number of any PCa and HGPCa tumors missed and unnecessary biopsies spared for base, base+PCA3 and base+lncRNA546 models in the threshold probability range of 10-40% in the PSA gray area cohort.**

| Probability cutoff, % | Model^β^ | Biopsies performed, No.(%) | Biopsies not performed, No.(%) | Unnecessary biopsies spared, No.(%) | Any PCa detected, No.(%) | Any PCa missed, No.(%) | NPV for any PCa, % | HGPCa detected, No.(%) | HGPCa missed, No.(%) | NPV for HGPCa, % |
| --- | --- | --- | --- | --- | --- | --- | --- | --- | --- | --- |
|  |  |  |  |  |  |  |  |  |  |  |
| 15 | base | 53(57.0) | 40(43.0) | 40(54.8) | 20(100.0) | 0(0.0) | 100.0 | 5(100.0) | 0(0.0) | - |
|  | base+PCA3 | 55(59.1) | 38(40.9) | 38(52.1) | 20(100.0) | 0(0.0) | 100.0 | 5(100.0) | 0(0.0) | - |
|  | base+lnc546 | 40(43.0) | 53(57.0) | 51(69.9) | 18(90.0) | 2(10.0) | 96.2 | 5(100.0) | 0(0.0) | 100.0 |
| 20 | base | 44(47.3) | 49(52.7) | 47(64.4) | 18(90.0) | 2(10.0) | 95.9 | 4(80.0) | 1(20.0) | 50.0 |
|  | base+PCA3 | 42(45.2) | 51(54.8) | 47(64.4) | 16(80.0) | 4(20.0) | 92.2 | 4(80.0) | 1(20.0) | 75.0 |
|  | base+lnc546 | 31(33.3) | 62(66.7) | 59(80.8) | 17(85.0) | 3(15.0) | 95.2 | 5(100.0) | 0(0.0) | 100.0 |
| 25 | base | 35(37.6) | 58(62.4) | 53(72.6) | 15(75.0) | 5(25.0) | 91.4 | 4(80.0) | 1(20.0) | 80.0 |
|  | base+PCA3 | 31(33.3) | 62(66.7) | 56(76.7) | 14(70.0) | 6(30.0) | 90.3 | 4(80.0) | 1(20.0) | 83.3 |
|  | base+lnc546 | 26(28.0) | 67(72.0) | 64(87.7) | 17(85.0) | 3(15.0) | 95.5 | 5(100.0) | 0(0.0) | 100.0 |
| 30 | base | 21(22.6) | 72(77.4) | 64(87.7) | 12(60.0) | 8(40.0) | 88.9 | 4(80.0) | 1(20.0) | 87.5 |
|  | base+PCA3 | 20(21.5) | 73(78.5) | 64(87.7) | 11(55.0) | 9(45.0) | 87.7 | 4(80.0) | 1(20.0) | 83.3 |
|  | base+lnc546 | 21(22.6) | 72(77.4) | 67(91.8) | 15(75.0) | 5(25.0) | 93.1 | 5(100.0) | 0(0.0) | 100.0 |
| 35 | base | 19(20.4) | 74(79.6) | 65(89.0) | 11(55.0) | 9(45.0) | 87.8 | 4(80.0) | 1(20.0) | 88.9 |
|  | base+PCA3 | 16(17.2) | 77(82.8) | 68(93.2) | 11(55.0) | 9(45.0) | 88.3 | 4(80.0) | 1(20.0) | 88.9 |
|  | base+lnc546 | 18(19.4) | 75(80.6) | 70(95.9) | 15(75.0) | 5(25.0) | 93.3 | 5(100.0) | 0(0.0) | 100.0 |
| 40 | base | 14(15.1) | 79(84.9) | 69(94.5) | 10(50.0) | 10(50.0) | 87.3 | 4(80.0) | 1(20.0) | 90.0 |
|  | base+PCA3 | 12(12.9) | 81(87.1) | 71(97.3) | 10(50.0) | 10(50.0) | 87.7 | 4(80.0) | 1(20.0) | 90.0 |
|  | base+lnc546 | 14(15.1) | 79(84.9) | 72(98.6) | 13(65.0) | 7(35.0) | 91.1 | 5(100.0) | 0(0.0) | 100.0 |

NPV=negative predictive value; HGPCa=high-grade prostate cancer (Gleason score≥7).

^β^The base model consists of age, volume, %fPSA and DRE.
